# Cancer Immunology and Immunotherapies: Mechanisms That Affect Antitumor Immune Response and Treatment Resistance

**DOI:** 10.3390/cancers13225655

**Published:** 2021-11-12

**Authors:** Xianda Zhao, Subbaya Subramanian

**Affiliations:** 1 Department of Surgery, University of Minnesota Medical School, Minneapolis, MN 55455, USA; 2 Masonic Cancer Center, University of Minnesota, Minneapolis, MN 55455, USA; 3 Center for Immunology, University of Minnesota, Minneapolis, MN 55455, USA

The past decade has seen immunotherapy rise to the forefront of cancer treatment. This Special Issue of *Cancers* aims to elaborate on the latest developments, cutting-edge technologies, and prospects in cancer immunology and immunotherapy. Seventeen exceptional studies, including original contributions and review articles, written by international scientists and physicians, primarily concerning the fields of tumor biology, cancer immunology, therapeutics, and drug development comprise the main body of this special issue. 

Over the last few years, an increasing understanding has emerged on molecular mechanisms that regulate the anti-tumor immune response and an exponential increase in the use of novel cancer immunotherapies in various cancer types. The field of Cancer immunology and Immunotherapies presents promising therapeutic opportunities for developing novel cancer treatments and improving patient survival outcomes. Chemotherapy is still used as a primary method for treatment, and the standard of care for many cancer types is relatively unselective and presents with the rapid development of treatment resistance. In contrast, cancer immunotherapies stimulate the antitumor immune response via the activation of lymphocytes that can recognize neoantigens, resulting in durable treatment response.

A successful antitumor immune response involves interactions between various cell types that coordinately function to prevent tumor cell proliferation or to effectively eradicate tumor cells. A coordinated functioning of the lymphoid and myeloid lineage cells is critical for killing tumor cells, and is performed by enhancing the activity of cytotoxic cells. Myeloid lineage cells, such as dendritic cells, provide tumor antigens to T cells and secrete cytokines that regulate the activation and function of cytotoxic cells. Despite the demonstrated successes of cancer immunotherapy, most patients do not respond, and the development of resistance has occurred in patients who initially respond to immunotherapies. Recent studies have uncovered novel immune escape mechanisms that affect immune cell infiltration, poor antigen presentation, and tumor intrinsic silencing of the immune response via cytokines and the release of immune suppressive exosomes [1]. Additional mechanisms of antitumor immune escape and immunotherapy resistance are continuously being discovered [2,3,4].

Based on these factors, significant attention has been directed towards the recent advances in cancer immunology [5,6,7,8,9,10]. In the past decades, the discovery of Programmed cell death protein 1 (PD-1) and the Cytotoxic T-lymphocyte–associated antigen 4 (CTLA-4) has helped to develop immune checkpoint blockade therapies. The articles by Yuan et al. [5] and Sobhani et al. [6] provide an overview and include recent findings on PD-1 and CTLA-4. The review article by Mehdi et al. [7] focuses on the role of methylation in manipulating cancer immunity. In addition to these general cancer immunology topics, reviews by Krishnamurthy et al. [8], Zheng et al. [9], and Marseglia et al. [10] summarize immune regulation in specific cancer types, such as hepatocellular carcinoma, triple-negative breast cancer, and uveal melanoma. 

The second series of articles mainly presents original work deciphering the novel regulatory mechanisms of cancer immunity. For the first time, our group (Wangmo et al. [11]) reported that Atypical Chemokine Receptor 4 (ACKR4) determines the migration of dendritic cells from tumor tissue to the tumor-draining lymph nodes. The loss of ACKR4 expression in tumor cells can affect the migration of dendritic cells and their retention in the tumor microenvironment, impairing T-cell priming in tumor-draining lymph nodes. This finding uncovers a novel mechanism that regulates dendritic cells’ migration from the tumor tissue, a critical factor in antigen presentation and in antitumor immune responses. Liang et al. [12] further contribute to the body of research regarding antigen-presenting cells. The authors performed an in-depth analysis of antigen-presenting cells in the human colorectal cancer microenvironment. Interestingly, they observed that antigen-presenting cells within distinct intratumoral and colonic milieus showed different functional statuses but were similarly responsive to induced T-cell activation. The third article in this section focuses on the bystander T-cells in cancers. In a hybrid study of bioinformatics and laboratory analyses, Gokuldass et al. [13] revealed a higher proportion of bystander CD8^+^ T cells in non-melanoma cancers than in melanoma cancers. This observation helps to establish a new theory to explain the different immune strengths of various tumors. In the context of innate immunity, Kaur et al. [14] reported on the function of CD16 receptors in both direct cytotoxicity and antibody-dependent cell cytotoxicity, making the use of these receptors as a cancer treatment seem promising.

The overarching objective of studying tumor immunity is to develop the next-generation cancer immunotherapies. In the third series of articles, several novel cancer immunotherapy strategies are proposed. Two original research articles from Jiang’s group [15,16] provide modified CAR T Cell therapies to treat malignant B-cell neoplasms and prostate cancer. Their modified CAR T cells are better directed to kill malignant B-cells, while sparing the CD19^+^HLA-C1^+^ healthy B Cells. The next study by Hsu et al. [17] developed a recombinant fusion IL15 protein composed of human IL15 (hIL15) and albumin-binding domain (hIL15-ABD) which has been successfully tested with anti-PD-L1 on CT26 murine colon cancer and B16-F10 murine melanoma models. Horn et al. [18] also reported on the use of IL15 as an agonist adjuvant for other cancer immunotherapies. Utilizing colon and mammary carcinoma models, the study showed that a recombinant adenovirus-based vaccine, targeting tumor-associated antigens with an IL-15 superagonist adjuvant is effective when combined with other immunotherapy regimens. This study also validated the idea that providing tumor-associated antigens as a vaccine helps to overcome immune checkpoint blockade resistance. Another feature in this issue is that we include a report on a new method called the ‘chemo-enzymatic conjugation approach’ (Bai et al. [19]) to generate bispecific antibodies (BiFab). Using this method, the authors produced BiFab^Her2/CD3^ and BiFab^CD20/CD3^ to conjugate both the target and effector cells (T-cells). These BiFabs demonstrated a strong considerable effect for inducing T-cell activation and killing target cancer cells upon conjugation. The BiFab^CD20/CD3^ also showed anti-tumor activity in vivo. 

The findings of Benajiba et al. [20] and Zwart et al. [21] highlight clinical observations relevant to cancer and immunology. Disseminated Kaposi’s sarcoma is usually treated by interferons, which is a type of immunotherapy. Benajiba et al. [20] performed a retrospective cohort study to evaluate global disease evolution and to identify the risk factors for systemic treatment initiation, including the use of interferons. They found that 41.2% of classic/endemic Kaposi’s sarcoma patients require systemic treatment. They also reported that the mean treatment-free time during the first five years following interferon is similar to that of chemotherapy. Lastly, Zwart et al. [21] contribute through a meta-analysis on immunosuppressive therapy after solid organ transplantation and on the development of cancers. Interestingly, the meta-analysis indicated that patients receiving cyclosporine A and Azathioprine after a solid organ transplant are at a higher risk than patients receiving other immunosuppressive drugs of developing certain types of cancers. 

In conclusion, the original research articles and reviews included in this special issue ensure that the key aspects of the next generation of cancer immunology and immunotherapy have been covered. We hope that the novel findings in these articles will inform the readers and provide useful references for developing next-generation cancer immunotherapies. 

## References

[1] Zhao X., Yuan C., Wangmo D., Subramanian S. (2021). Tumor-Secreted Extracellular Vesicles Regulate T-Cell Costimulation and Can Be Manipulated To Induce Tumor-Specific T-Cell Responses. Gastroenterology.

[2] Zhao X., Wangmo D., Robertson M., Subramanian S. (2020). Acquired Resistance to Immune Checkpoint Blockade Therapies. Cancers.

[3] Zhao X., Subramanian S. (2018). Oncogenic pathways that affect antitumor immune response and immune checkpoint blockade therapy. Pharmacol. Ther..

[4] Zhao X., Subramanian S. (2017). Intrinsic Resistance of Solid Tumors to Immune Checkpoint Blockade Therapy. Cancer Res..

[5] Yuan Y., Adam A., Zhao C., Chen H. (2021). Recent Advancements in the Mechanisms Underlying Resistance to PD-1/PD-L1 Blockade Immunotherapy. Cancers.

[6] Sobhani N., Tardiel-Cyril D., Davtyan A., Generali D., Roudi R., Li Y. (2021). CTLA-4 in Regulatory T Cells for Cancer Immunotherapy. Cancers.

[7] Mehdi A., Rabbani S. (2021). Role of Methylation in Pro- and Anti-Cancer Immunity. Cancers.

[8] Krishnamurthy S., Gilot D., Ahn S.B., Lam V., Shin J.-S., Guillemin G.J., Heng B. (2021). Involvement of Kynurenine Pathway in Hepatocellular Carcinoma. Cancers.

[9] Zheng H., Siddharth S., Parida S., Wu X., Sharma D. (2021). Tumor Microenvironment: Key Players in Triple Negative Breast Cancer Immunomodulation. Cancers.

[10] Marseglia M., Amaro A., Solari N., Gangemi R., Croce E., Tanda E., Spagnolo F., Filaci G., Pfeffer U., Croce M. (2021). How to Make Immunotherapy an Effective Therapeutic Choice for Uveal Melanoma. Cancers.

[11] Wangmo D., Premsrirut P.K., Yuan C., Morris W.S., Zhao X., Subramanian S. (2021). ACKR4 in Tumor Cells Regulates Dendritic Cell Migration to Tumor-Draining Lymph Nodes and T-Cell Priming. Cancers.

[12] Liang F., Rezapour A., Szeponik L., Alsén S., Wettergren Y., Lindskog E.B., Quiding-Järbrink M., Yrlid U. (2021). Antigen Presenting Cells from Tumor and Colon of Colorectal Cancer Patients Are Distinct in Activation and Functional Status, but Comparably Responsive to Activated T Cells. Cancers.

[13] Gokuldass A., Draghi A., Papp K., Borch T.H., Nielsen M., Westergaard M.C.W., Andersen R., Schina A., Bol K.F., Chamberlain C.A. (2020). Qualitative Analysis of Tumor-Infiltrating Lymphocytes across Human Tumor Types Reveals a Higher Proportion of Bystander CD8(+) T Cells in Non-Melanoma Cancers Compared to Melanoma. Cancers.

[14] Kaur K., Safaie T., Ko M.-W., Wang Y., Jewett A. (2021). ADCC against MICA/B Is Mediated against Differentiated Oral and Pancreatic and Not Stem-Like/Poorly Differentiated Tumors by the NK Cells; Loss in Cancer Patients due to Down-Modulation of CD16 Receptor. Cancers.

[15] He C., Zhou Y., Li Z., Farooq M.A., Ajmal I., Zhang H., Zhang L., Tao L., Yao J., Du B. (2020). Co-Expression of IL-7 Improves NKG2D-Based CAR T Cell Therapy on Prostate Cancer by Enhancing the Expansion and Inhibiting the Apoptosis and Exhaustion. Cancers.

[16] Tao L., Farooq M.A., Gao Y., Zhang L., Niu C., Ajmal I., Zhou Y., He C., Zhao G., Yao J. (2020). CD19-CAR-T Cells Bearing a KIR/PD-1-Based Inhibitory CAR Eradicate CD19(+)HLA-C1(-) Malignant B Cells While Sparing CD19(+)HLA-C1(+) Healthy B Cells. Cancers.

[17] Hsu F.-T., Liu Y.-C., Tsai C.-L., Yueh P.-F., Chang C.-H., Lan K.-L. (2021). Preclinical Evaluation of Recombinant Human IL15 Protein Fused with Albumin Binding Domain on Anti-PD-L1 Immunotherapy Efficiency and Anti-Tumor Immunity in Colon Cancer and Melanoma. Cancers.

[18] Horn L., Fousek K., Hamilton D., Hodge J., Zebala J., Maeda D., Schlom J., Palena C. (2021). Vaccine Increases the Diversity and Activation of Intratumoral T Cells in the Context of Combination Immunotherapy. Cancers.

[19] Bai X., Liu W., Jin S., Zhao W., Xu Y., Zhou Z., Chen S., Pan L. (2021). Facile Generation of Potent Bispecific Fab via Sortase A and Click Chemistry for Cancer Immunotherapy. Cancers.

[20] Benajiba L., Lambert J., La Selva R., Cochereau D., Baroudjian B., Roux J., Le Goff J., Pages C., Battistella M., Delyon J. (2021). Systemic Treatment Initiation in Classical and Endemic Kaposi’s Sarcoma: Risk Factors and Global Multi-State Modelling in a Monocentric Cohort Study. Cancers.

[21] Zwart E., Yüksel E., Pannekoek A., de Vries R., Mebius R., Kazemier G. (2021). De Novo Carcinoma after Solid Organ Transplantation to Give Insight into Carcinogenesis in General-A Systematic Review and Meta-Analysis. Cancers.

